# Resonance of graphene nanoribbons doped with nitrogen and boron: a molecular dynamics study

**DOI:** 10.3762/bjnano.5.84

**Published:** 2014-05-27

**Authors:** Ye Wei, Haifei Zhan, Kang Xia, Wendong Zhang, Shengbo Sang, Yuantong Gu

**Affiliations:** 1 MicroNano System Research Center, Key Lab of Advanced Transducers and Intelligent Control System of the Ministry of Education & College of Information Engineering, Taiyuan University of Technology, Taiyuan 030024, Shanxi, China; 2 School of Chemistry, Physics and mechanical Engineering, Queensland University of Technology, Brisbane, QLD 4109, Australia

**Keywords:** dopant, graphene, molecular dynamics simulation, natural frequency, quality factor, resonance

## Abstract

Based on its enticing properties, graphene has been envisioned with applications in the area of electronics, photonics, sensors, bio-applications and others. To facilitate various applications, doping has been frequently used to manipulate the properties of graphene. Despite a number of studies conducted on doped graphene regarding its electrical and chemical properties, the impact of doping on the mechanical properties of graphene has been rarely discussed. A systematic study of the vibrational properties of graphene doped with nitrogen and boron is performed by means of a molecular dynamics simulation. The influence from different density or species of dopants has been assessed. It is found that the impacts on the quality factor, *Q*, resulting from different densities of dopants vary greatly, while the influence on the resonance frequency is insignificant. The reduction of the resonance frequency caused by doping with boron only is larger than the reduction caused by doping with both boron and nitrogen. This study gives a fundamental understanding of the resonance of graphene with different dopants, which may benefit their application as resonators.

## Introduction

Graphene has drawn intensive interest since its discovery in 2005 [1]. It has been reported to have supreme stiffness (Young’s modulus ≈ 1 TPa), very high electron mobility, electrical and thermal conductivity, optical absorption as well as many other excellent properties [2–3]. These properties of graphene open up huge potential applications in the area of electronics, photonics, composite materials, energy generation and storage, sensors, and biomedicine or bio-applications [3–5]. A great effort has been devoted to modify the properties of graphene to facilitate these promising applications, which leads to a variety of graphene derivatives or hybrid structures.

Doping as one of the common approaches to manipulate the properties of nanomaterials has received wide applications in the synthesis of graphene derivatives. There are two typical doping schemes. One is the so-called electrical doping, which does not change the lattice structure or chemical composition of the graphene, such as the absorption of a gas or a metal (e.g., Ti, Fe, Pt). The other is chemical doping, which introduces substitutional atoms to graphene, e.g., nitrogen (N) [6], boron (B), sulphur (S) and silicon (Si) [7]. By either electrical or chemical doping, one can significantly alter the electronic and quantum transport properties of graphene. Such doped graphene is envisioned with exciting applications as high-performance FET devices [8], and metal-free electrocatalyst for oxygen reduction fuel cells [9]. In addition to doping, various graphene derivatives have also been synthesised through chemical functionalization with hydroxy and methyl groups or hydrogen [10], the decoration with quantum dots [11], noble metal nanoparticles (NPs) [12], or complex biomolecular structures [13–14]. A number of works have been conducted to investigate the properties of graphene derivatives, which however usually focus on their electrical or chemical properties, leaving their mechanical properties being rarely discussed. For instance, based on the Raman spectroscopy, the interactions between metal NPs (such as Au, Ag, Pt and Pd) and graphene were examined [15–16]. The structural and electrical properties of Pt, Fe, and Al NPs adsorbed on monovacancy-defective graphene were explored by density functional theory (DFT) calculations [17–18]. To accommodate different applications of graphene derivatives, a comprehensive understanding of their mechanical properties is crucial. For instance, graphene is proposed to build the ultimate of two dimensional nanoelectromechanical systems (as a resonator) owing to its ultrasensitive detection of mass, force and pressure [19].

Therefore, in this paper, we will discuss extensively on the vibration properties of graphene nanoribbons (GNRs) with different dopants. The study will be carried out by large-scale molecular dynamics (MD) simulations. Both perfect and defective (with initial vacancies) GNRs doped with boron and nitrogen will be considered.

## Numerical implementation

Based on large-scale molecular dynamics (MD) simulations, a series of vibrational studies of the GNRs with different dopants have been conducted. The testing samples with a uniform sample size of about 2 × 10 nm^2^ and armchair edges along the length direction were either doped with B or both B and N atoms. According to previous experimental work [20–22], different densities of dopants have been adopted to build the testing models. Three groups of samples have been tested based on three different GNRs, e.g., a perfect GNR, a defective GNR with two vacancies, and a defective GNR with four vacancies. Each group contains two subgroups, one of which is only doped with boron and the other is doped with both boron and nitrogen (doping with only nitrogen has already been investigated in our earlier work [23], hence it will not be considered herein). The different dopants were randomly distributed in the GNR. Since the N–N single bond is chemically unstable due to the low bonding energy, such bonds will not exist in doped models. To ensure a reasonable comparison, the sample with a higher density of dopants contains all the dopant positions in the model with a lower doping percentage.

To describe the atomic interactions between carbon atoms, the commonly utilised reactive empirical bond order (REBO) potential [24] was employed, which gives a good representation for the binding energies and elastic properties of carbon nanotubes and graphene [25]. In general, the REBO potential is given as

**Figure 1:**
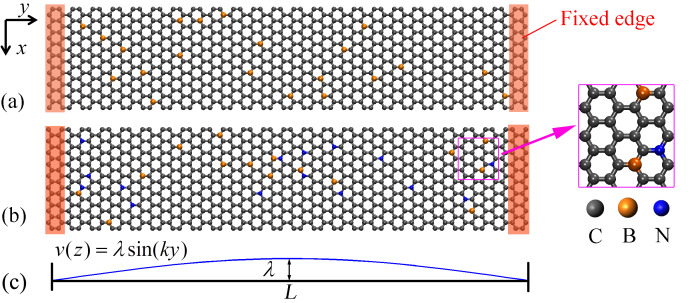
(a) A perfect GNR with 3% B-dopant. (b) A perfect GNR with 1.5% B- and 1.5% N-dopant. (c) Velocity excitation profile. The regions highlighted in red in (a) and (b) represent the fixed edges.

[1]
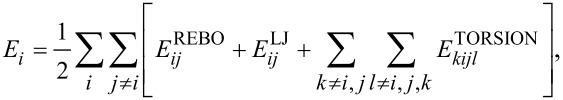


where, *E*^REBO^ represents the short-distance C–C interaction, *E*^LJ^ depicts a longer-distance C–C interaction in the form of a typical Lennard-Jones (LJ) potential. The last term describes the dihedral-angle preferences in hydrocarbon configurations. For the other atomic interactions induced by the dopant atoms (i.e., C–B, C–N, and B–N), a typical Tersoff potential [26] was adopted. For each simulation, the conjugate gradient algorithm was firstly applied to relax the sample to a minimum energy state. Afterwards, the sample was equilibrated under a Nose–Hoover thermostat at 5 K. Finally, the graphene layer was actuated by applying a sinusoidal velocity excitation *v*(*z*) = λ·sin(*ky*) along the z-axis, where λ is the actuation amplitude (here 0.6 Å), and *k* = π/*L* (here *L* is the effective length of the graphene layer, which excludes the two fixed edges, see Figure 1). In the end, a microcanonical (*NVE*) ensemble was applied to simulate the free vibration of the system. The equations of moving atoms are integrated over time by using a Velocity Verlet algorithm [27]. In order to account for the spurious edge modes of the GNRs, a non-periodical boundary condition was applied along any direction during the whole simulation.

## Results and Discussions

**Figure 2:**
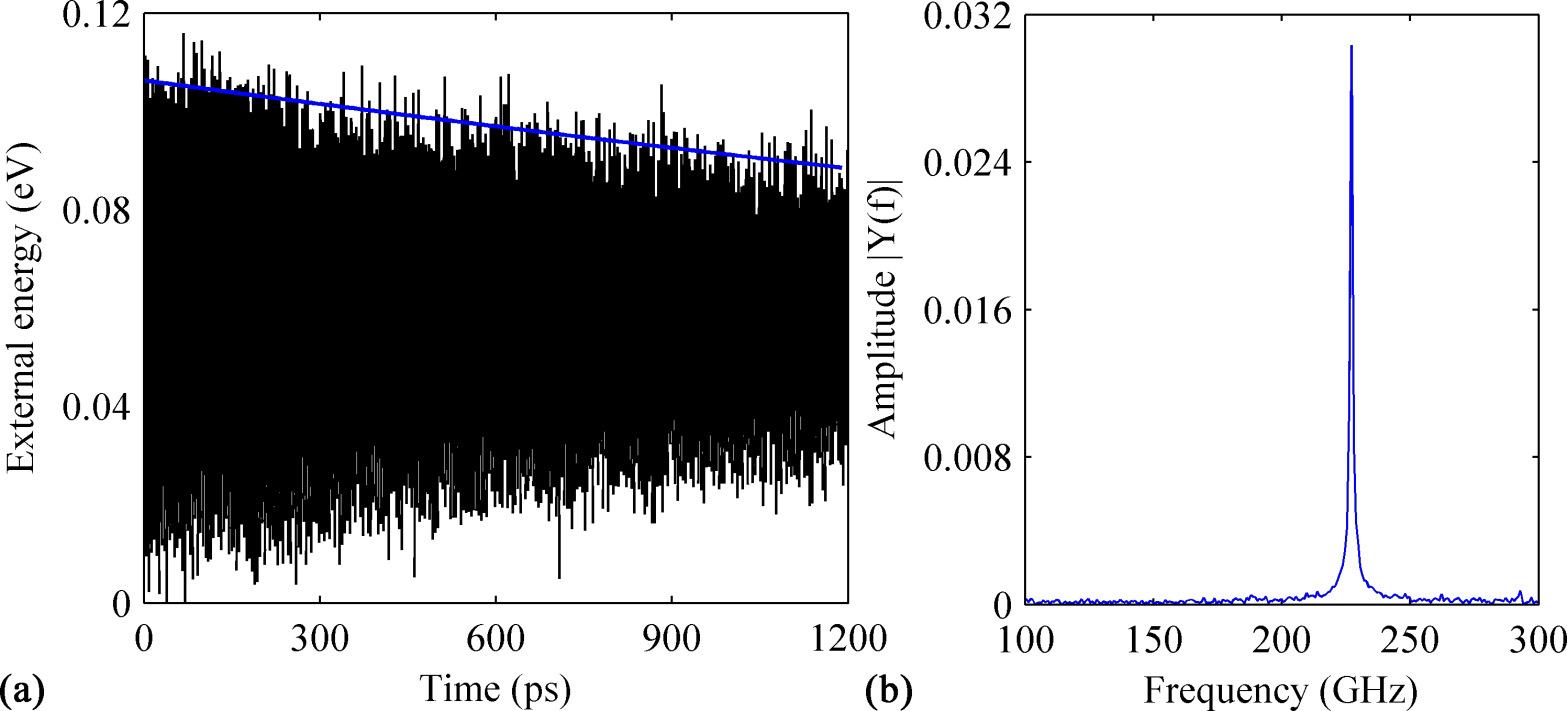
(a) Variation in time of the external energy obtained from a perfect GNR. (b) The corresponding frequency spectrum obtained.

To acquire the impact from the dopants on the resonance properties of graphene, emphasis has been put on the quality factor, *Q*, and resonance frequency. These values are calculated following the commonly utilized estimation schemes by previous researchers [28–29]. That is, *Q* is defined as the ratio between the total system energy and the average energy loss in one radian at the resonant frequency [30], i.e., *Q* = 2π*E*/Δ*E*, where *E* is the total energy of the vibrating system and Δ*E* is the energy dissipated by damping during one cycle of vibration. The value of *Q* is assumed as to be constant during vibration, which gives a relation between the maximum energy (*E_n_*) and the initial maximum energy (*E*_0_) as *E*_n_ = *E*_0_(1 − 2π/*Q*)*^n^* after *n* vibration cycles [31]. Since an energy-preserving *NVE* ensemble is assumed during vibration and the simulation is under vacuum conditions, the damping will result from intrinsic loss only. Therefore, the loss of potential energy must be converted to kinetic energy. Thus, the change of the external energy over time will be tracked for the calculation of *Q*. The external energy is defined as the difference of the potential energy before and after the initial excitation is applied to the testing sample [32]. Regarding the resonance frequency, a discrete Fourier transform will be applied [33]. For comparison, the vibration properties for the prefect GNR are firstly assessed. As shown in Figure 2a, the amplitude of the external energy decreases linearly with the increase of simulation time, with *Q* being estimated to be 4660. The corresponding frequency spectrum of the GNR is derived from fast Fourier transformation. As shown in Figure 2b, the natural frequency of the external energy is about 228 GHz. As the energy is a square function of the velocity, i.e., the natural frequency of the GNR is half of the external energy frequency, or 114 GHz.

### Perfect GNRs with dopants

#### Influence of B-dopant

**Figure 3:**
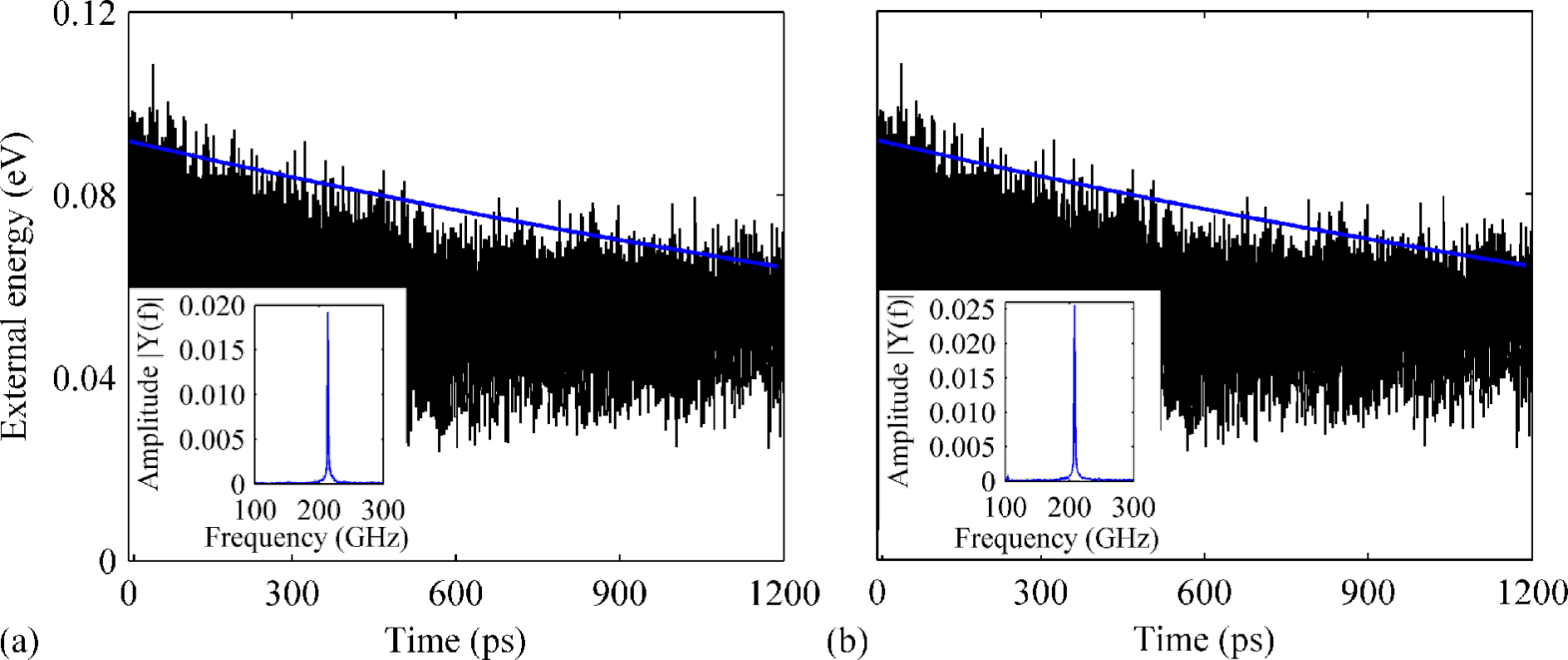
Variation of history of the external energy over time for a perfect GNR with B-dopant densities of (a) 0.76% and (b) 1.89%. The insets show the corresponding frequency spectra.

At the beginning, we consider the impact of B-dopants on the resonance properties of a perfect GNR. Six testing samples were established with a doping ratio ranging from 0.25% to 2.40%. Generally, it is found that the presence of different densities of B-dopant reduces *Q* compared to that of pristine perfect GNR (shown in Figure 2a). In particular, the GNR with a B-dopant densities of 0.38%, 0.76%, 1.14% and 1.64% have a similar *Q* of around 2200, which means a reduction of more than 50%. The results obtained from the GNR with 0.76% B-dopant are illustrated in Figure 3a. Clearly, the amplitude of the external energy is found to decrease from about 0.10 to about 0.07 eV over the simulation time, which leads to Q ≈ 2260. From the corresponding frequency spectrum, the resonance frequency is estimated to be 107 GHz. Interestingly, the highest *Q* is found for the GNR with 1.89% B-dopant (Q ≈ 3070, about 34% reduction), followed by the GNR with 2.65% B-dopant (Q ≈ 2770). This phenomenon suggests that there is no correlation between the reduction of *Q* and the density of B-dopant (within the considered maximum density of 2.65%). Comparing with the results presented in Figure 2a, a much slower energy dissipation is found for the GNR with 1.89% B-dopant (see Figure 2b). We note that, although the GNR with a higher density of B-dopant might have a higher *Q*, the resonance frequency appears to have a consistent trend to decrease when the B-content is increased. It is concluded from all considered six cases that the reduction of *Q* does not necessarily increase with increasing density of dopants.

#### Influence of both B- and N-dopants

We then consider the GNR with both B- and N-dopants. Again, we consider six different cases, with the percentages of dopant atoms ranging from 0.26% to 2.78%, in which B and N share the same density, namely half of the total percentage. Strikingly, the GNR with 0.26% of B- and N-dopants appear to have an enhanced *Q-*factor of about 9050, which is twice of that observed from the pristine GNR. Except this case, the rest five samples exhibit apparent deteriorating vibration behavior.

**Figure 4:**
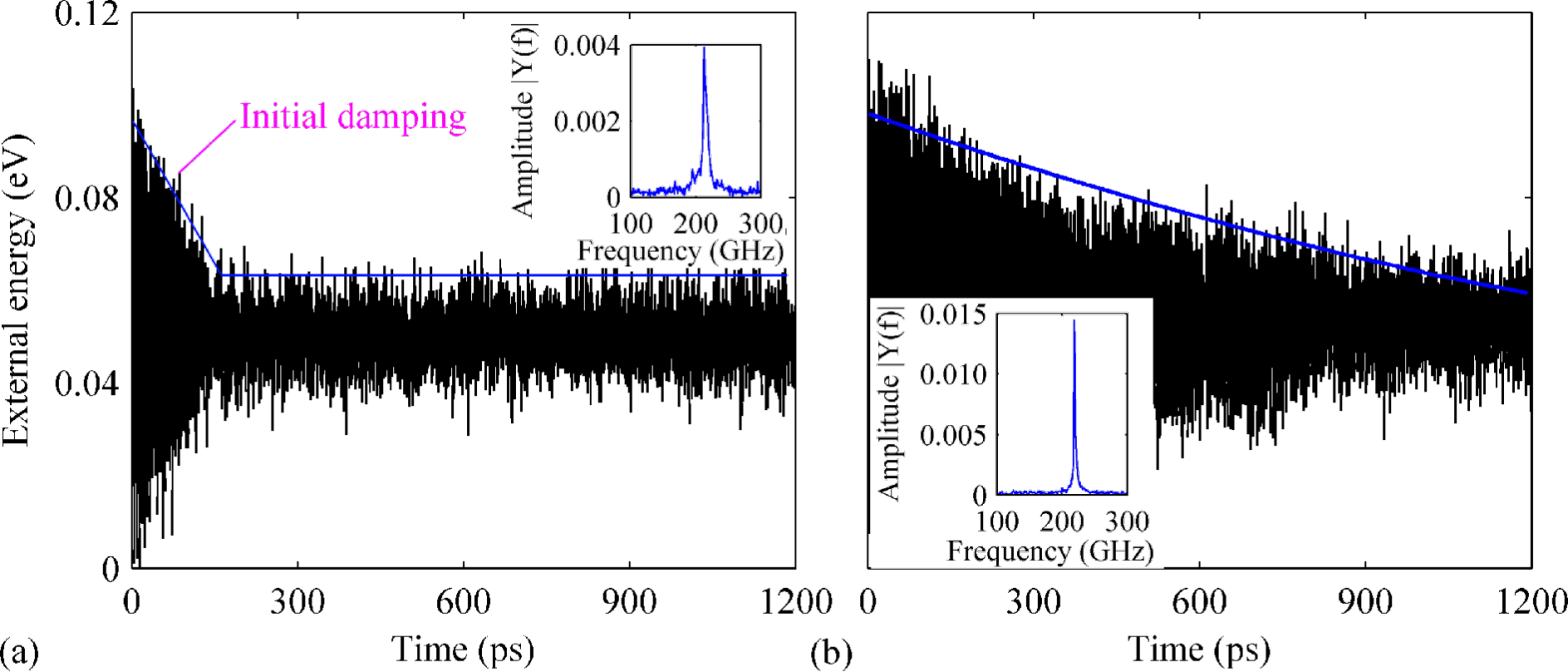
Variation of the external energy over time for a perfect GNR with B- and N-dopants. The total density of dopants is (a) 1.27% and (b) 1.65%. The insets show the corresponding frequency spectra.

Particularly, the profiles of the external energy of the GNRs with a density of 1.27% and 2.28% differ greatly from that of other cases, i.e., the amplitude does not show a consistent linear decrease fashion. As shown in Figure 4a, the external energy is observed to experience a sharp dissipation from 0.10 to 0.06 eV within 150 ps of simulation time. Afterwards, it fluctuates around 0.06 eV with no obvious dissipation. From the frequency spectrum, the resonance frequency is estimated to be 106 GHz. For the GNRs with a density of 0.76%, 1.65% and 2.78%, a similar progression of the external energy is found. As shown in Figure 4b, the external energy of the GNR with 1.65% of dopants decreases quickly from 0.10 to 0.03 eV after 1200 ps of simulation time. A low *Q* of about 1610 is estimated with a corresponding natural frequency of 109 GHz. In all, although different densities of dopants influence the value of Q differently, the resonance frequency generally decreases with an increasing of the dopant density.

### Defective GNRs with two vacancies

Experimental results show that defects normally exist in GNRs. These defects can be grain boundaries, oxidations or vacancies [34–35]. Specifically, for GNRs with vacancies, researchers reported three different types of nitrogen doping, namely graphitic N, pyridine-like N and pyrrole-like N depending on their locations. Therefore, in the following, we consider the vibration properties of defective GNRs (with either two or four vacancies) with different dopants. We start from the GNR with two vacancies, discussions are given as below.

#### Influence of B-dopant

**Figure 5:**
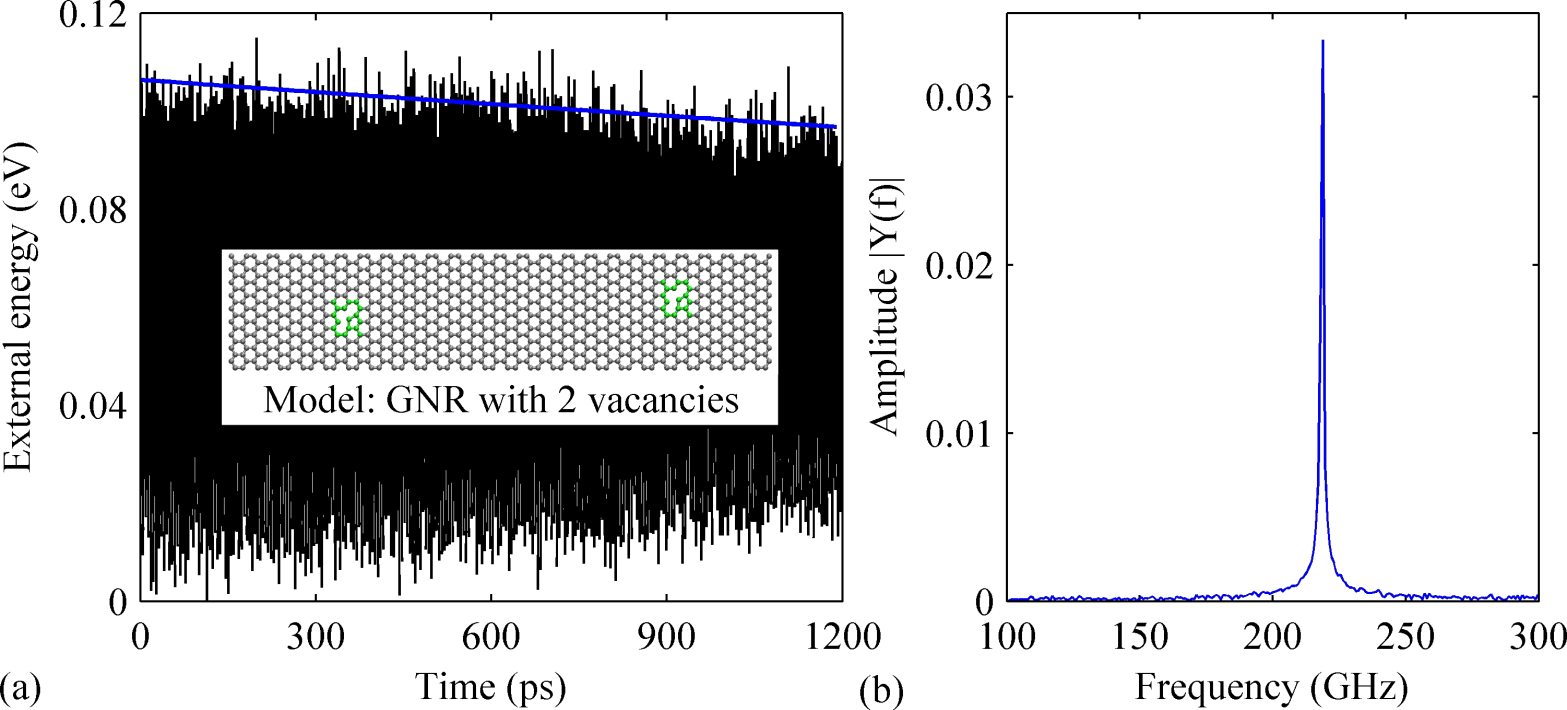
(a) Variation of the external energy over time obtained for a pristine GNR with two vacancies. The inset shows the corresponding model of the GNR. The regions highlighted in green represent the vacancies. (b) The corresponding frequency spectrum.

Similarly, seven different samples (including the pristine defective GNR) have been studied with a dopant density range of 0.38–2.40%. Random locations are adopted for the two vacancies as shown in the inset of Figure 5a. The MD results from the pristine defective GNR are depicted in Figure 5. Comparing with the results from the pristine perfect GNR (given in Figure 2a), the amplitude of the external energy shows an even smaller decrease pattern (from about 0.11 to 0.10 eV), which signifies a larger *Q* of about 8630. While an obvious decrease of the resonance frequency of about 4% is observed.

**Figure 6:**
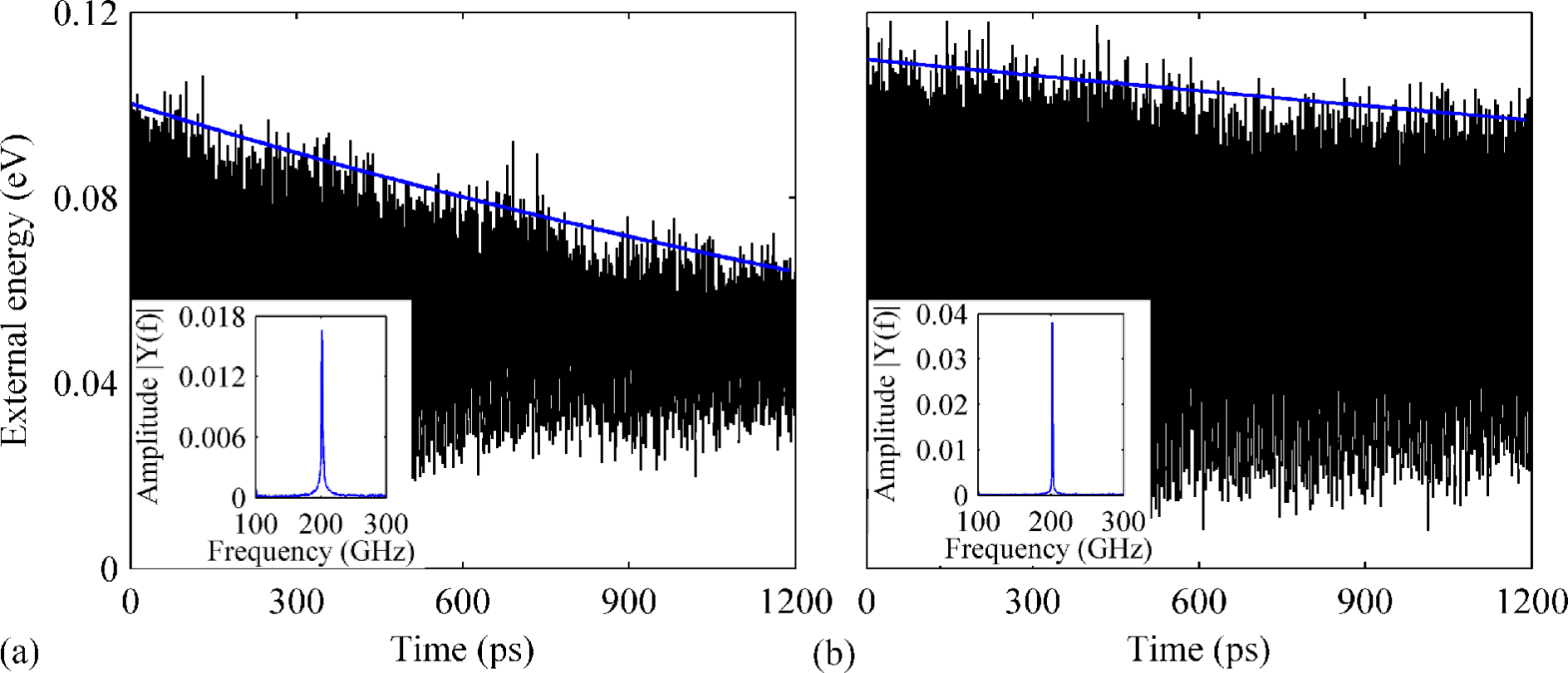
Variation of the external energy over time for a defective GNR (two vacancies) with B-dopant. The density of the dopant is (a) 1.64% and (b) 1.89%. The insets show the corresponding frequency spectra.

Consistent with the phenomena observed from perfect GNR with B-dopant (see above), the existence of B-dopant also induces an evident reduction to of *Q*. All doped cases except the one with 1.89% B-dopant show a greatly deteriorated *Q* with a reduction of over 70%. The smallest *Q* is found for the case containing 1.64% B-dopant. As illustrated in Figure 6a, the amplitude of the external energy decreases sharply from 0.10 to 0.07 eV within the 1200 ps simulation time. The value of *Q* is estimated as low as 1700, which is a reduction of over 80% compared to that of the pristine defective GNR. Besides, a relatively high *Q* is also found for the defective GNR with 1.89% B-dopant. As illustrated in Figure 6b, the amplitude of the external energy decreases slowly from 0.15 to 0.9 eV after 1200 ps and *Q* is calculated to be 6000. The fact the samples with higher densities of dopants exhibit higher *Q* further suggest that there is no correlation between the amount of dopants and the value of *Q*.

#### Influence of both B- and N-dopants

**Figure 7:**
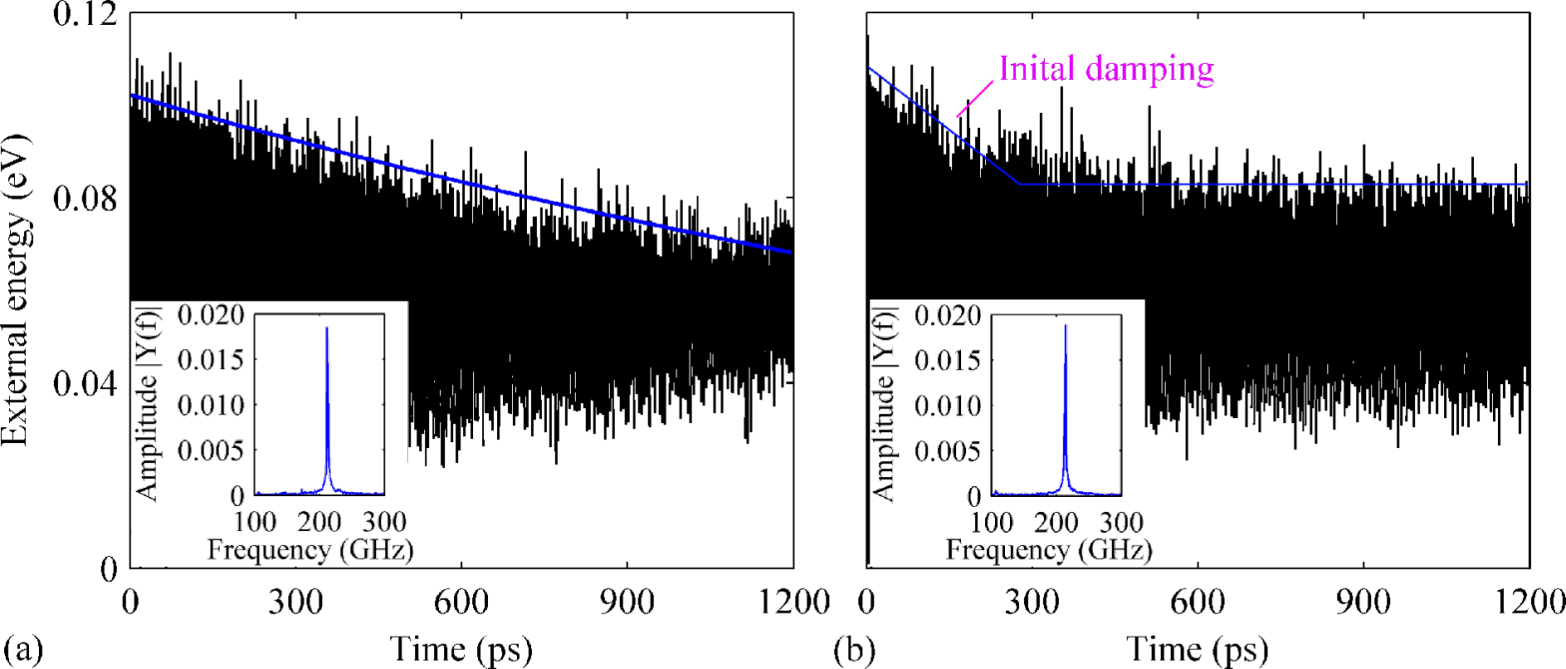
Variation of the external energy over time for the defective GNR (two vacancies) with both B- and N-dopants. The total dopant densities are (a) 0.89% and (b) 2.02%. The insets show the corresponding frequency spectra.

The influence of both B- and N-dopants on the resonance properties of the defective GNR are examined next. Different dopant densities including 0.38%, 0.88%, 1.27%, 1.77%, 2.02% and 2.53% are considered. Overall, the influence from the different densities of dopants varies greatly. For the defective GNR with 0.38% B- and N-dopants, a very high *Q* of about 8300 is observed, while for the case with 0.88% dopants, an extremely low *Q* of about 1980 is detected. Figure 7a depicts the results obtained from the case with 0.38% dopants. A fast energy dissipation is observed, with the resonance frequency being estimated to be 109 GHz (the same as that obtained from the pristine defective GNR).

Different behaviors are also found for the other two samples with dopant densities of 1.77% and 2.02%. The results from the case with 2.02% dopants are given in Figure 7b. As clearly seen, the external energy exhibit a sharp initial damping from 0.11 to ca. 0.09 eV at the early stage of vibration (within 300 ps). Afterwards, it saturates around 0.09 eV. The corresponding resonance frequency is estimated to be 107 GHz.

### Defective GNR with four vacancies

#### Influence of B-dopant

**Figure 8:**
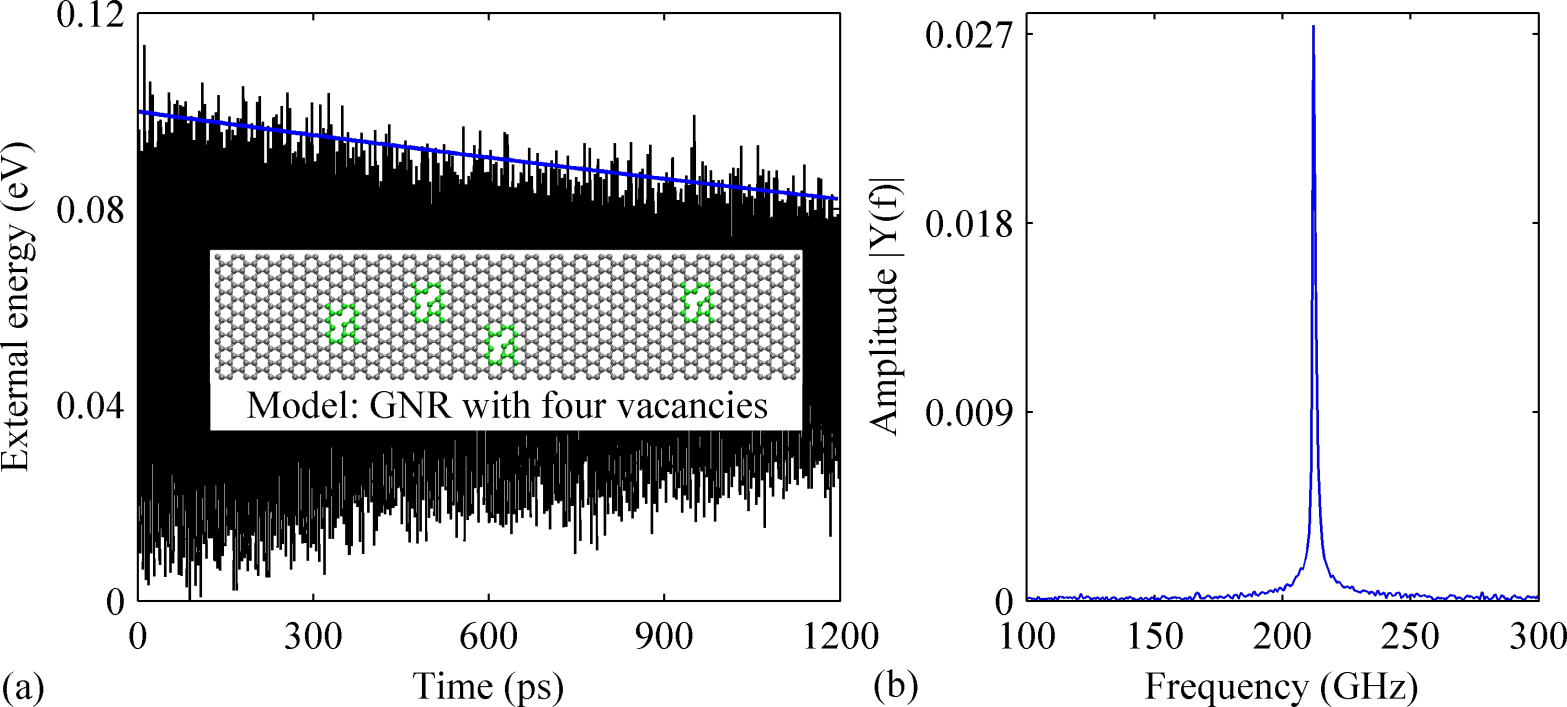
(a) Time history of the external energy obtained from pristine defective GNR with four vacancies. The inset shows the corresponding model of GNR with four vacancies. The regions highlighted in green represent the vacancies. (b) The corresponding frequency spectrum.

To further examine the influence of a combination of vacancies and dopants, we establish another GNR model with four randomly distributed vacancies (see the inset of Figure 8a). The simulation results obtained from the pristine case are presented in Figure 8. Compared to the GNR with two vacancies, a lower *Q* and a lower resonance frequency are observed, about 4080 and about 106 GHz, respectively.

**Figure 9:**
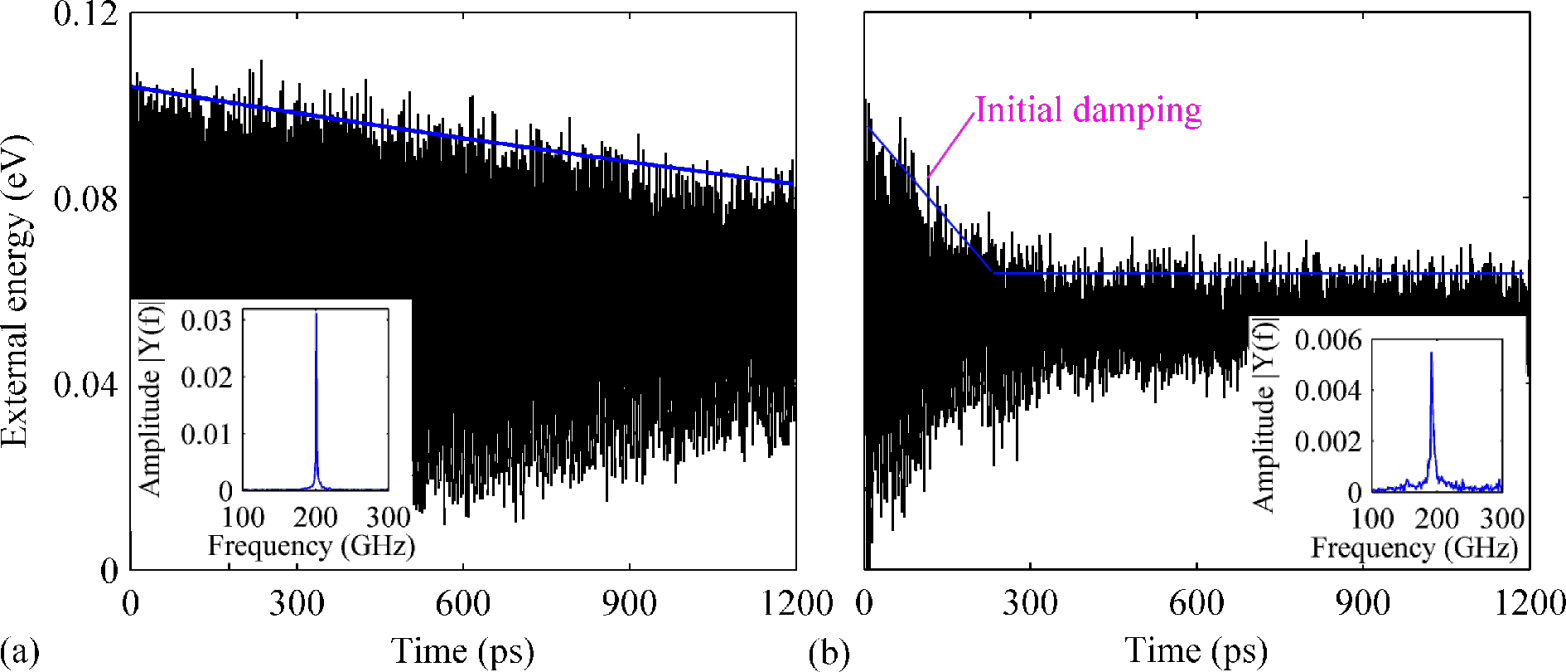
Variation over time of the external energy of the defective GNR with four vacancies and B-dopant densities of (a) 0.76% and (b) 2.40%. The insets show the corresponding frequency spectra.

Based on the above results, we then compare the resonance properties of the GNR (with four vacancies) with the presence of B-dopants (density ranges from 0.26% to 2.40%). Comparing with all previous cases, a relatively smaller degradation is found for *Q*. The smallest *Q*, which is about 2620, is observed in the case with 1.77% B-dopants, a 35% reduction comparing with that of the pristine GNR in Figure 8a. For densities of B-dopant density ranging from 0.26% to 1.90%, a similar variation over time of the external energy is observed. As shown in Figure 9a, the amplitude of the external energy for the case with 0.76% B-dopant decreases linearly from 0.10 to 0.08 eV after 1200 ps. A non-uniform linear decrease fashion of the external energy is also detected in the defective GNR with 2.40% B-dopant. Figure 9b shows a fast energy dissipation at the beginning of the vibration and the external energy saturates at around 0.06 eV. Besides of the evident impacts of the B-doping on *Q*, a continuous reduction of the resonance frequency is also observed with increasing B-dopant percentage.

#### Influence of both B- and N-dopants

**Figure 10:**
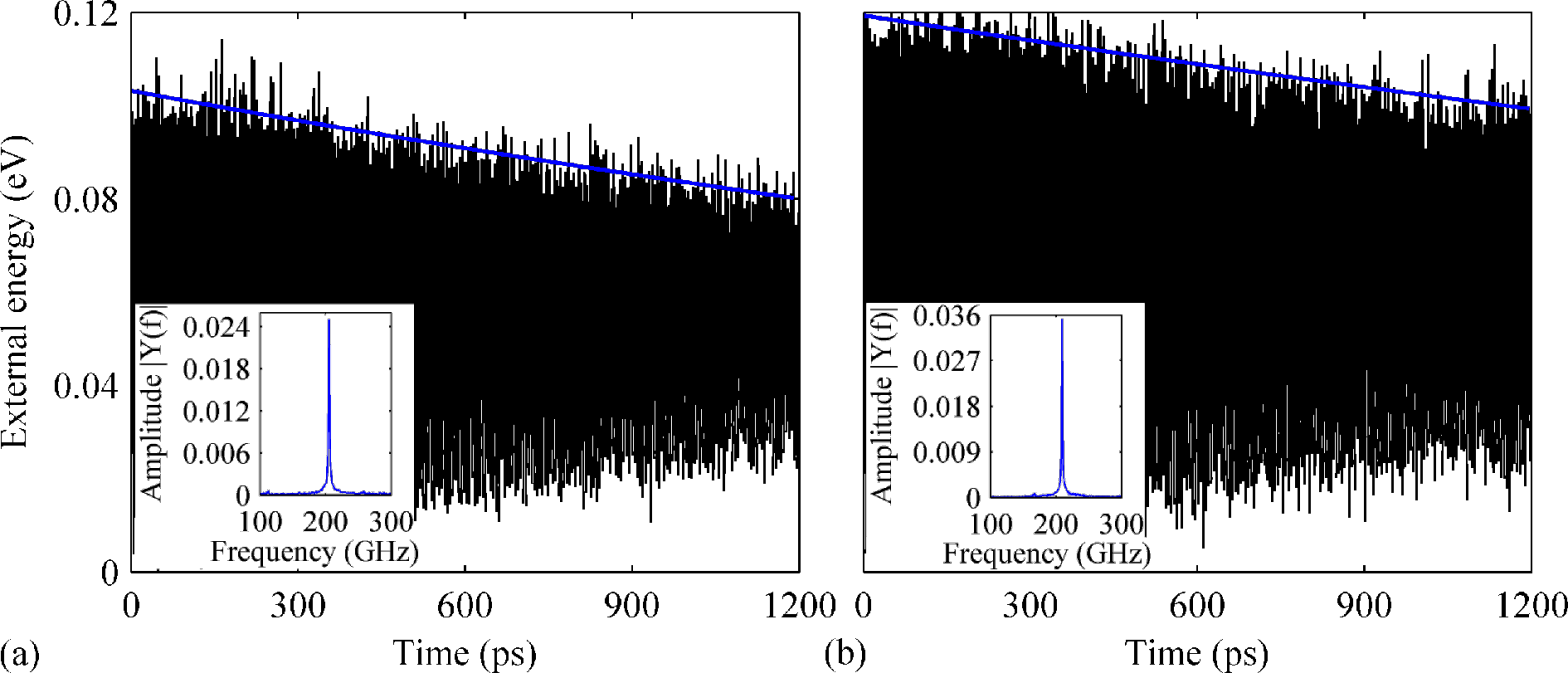
Variation over time of the external energy for the defective GNR (four vacancies) with both B- and N-dopants. The total densities of dopants are (a) 0.76% and (b) 1.76%. The insets show the corresponding frequency spectra.

In the end, we investigate the resonance properties of the GNR with four vacancies and B- and N-dopants. Considered dopant’s density ranges from 0.26% to 2.40%. Interestingly, only the case with 0.76% of dopants is found to exhibit a decreased *Q*, which is around 3050 (Figure 10a). All other samples are found to have an enhanced *Q*. As seen in Figure 10b, the amplitude of the external energy of the case with 1.76% of dopants linearly decreases from 0.12 to 0.10 eV with the *Q* being estimated to be 4290 (a slight increase about 5% comparing with that of the pristine defective GNR shown in Figure 8a).

**Figure 11:**
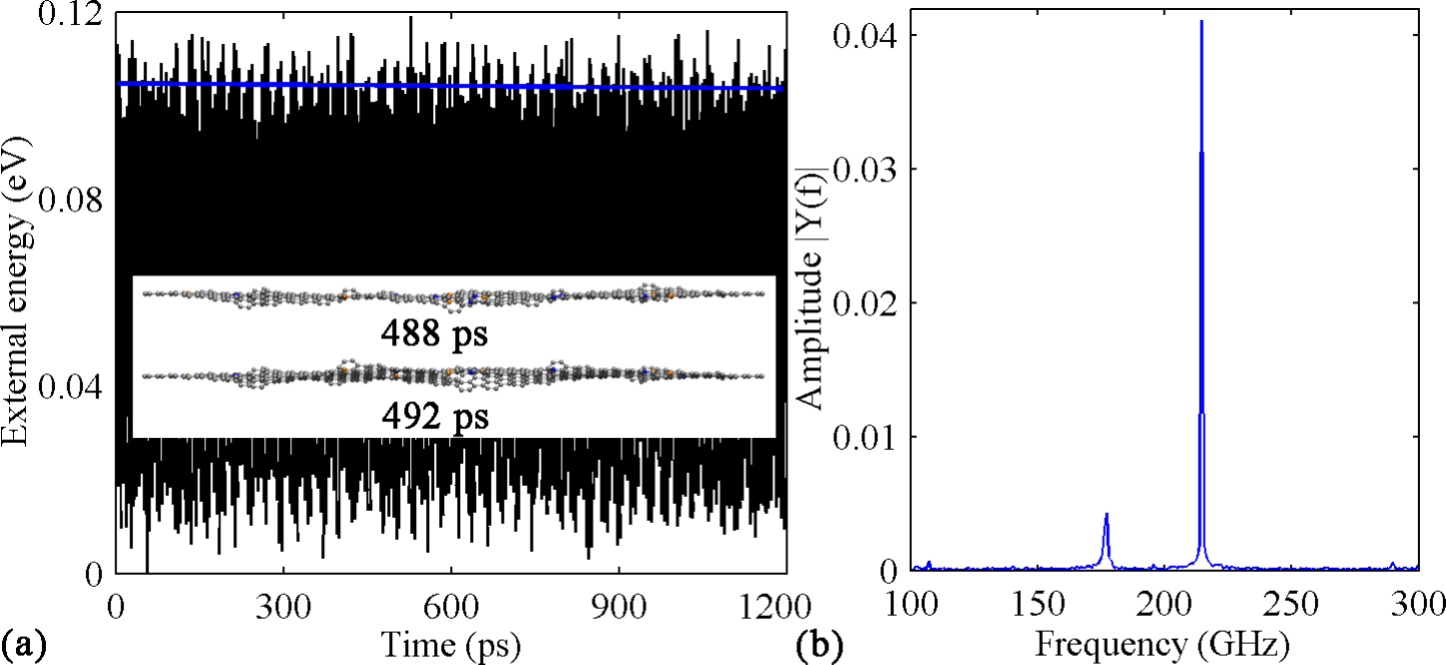
Results of the defective GNR (four vacancies) with 1.20% B- and 1.20% N-dopant. (a) Variation over time of the external energy. The inset shows the sample at the simulation time of 488 and 492 ps. (b) The corresponding frequency spectrum.

The most significantly increased *Q* is observed for the case with 2.40% dopant density. As shown in Figure 11a, no obvious energy dissipation is found, which results in a *Q-*factor as high as 79020. The corresponding frequency spectrum reveals that there are two resonance modes existing. Close inspection of the atomic configurations of the sample shows that the GNR is vibrating along both lateral and length directions (see the insets in Figure 11a). It is evident that the vibration behavior is dominated by the vertical vibration, as indicated by the extremely small amplitude of the translational vibration comparing with that of the vertical vibration in Figure 11b. To further justify this observation, a smaller excitation amplitude (0.4 Å/ps) has been tested, for which we still find the co-existence of the vertical and translation vibration modes. A similar phenomenon is also observed for the case with a dopant density of 1.90%. It is concluded that for the GNR with four vacancies, a higher density of dopants will make the translational vibration mode much easier to be excited.

**Figure 12:**
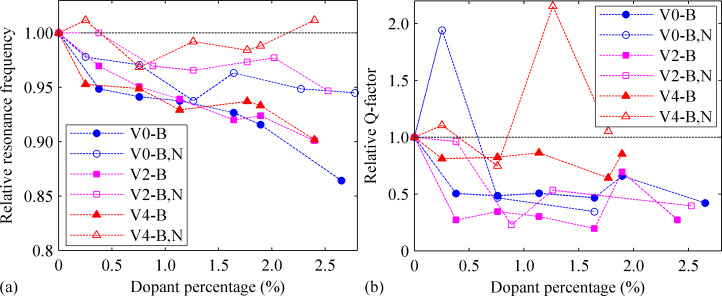
(a) Comparisons of the relative natural frequency among all studied samples. (b) Comparisons of the relative *Q* factor among all studied samples. V0-B represents the group of a perfect GNR with B-dopant, V0-B,N represents the group of a perfect GNR with both B- and N-dopants; V2-B represents the two vacancies-GNR with B-dopants, V2-B,N represents the two vacancies-GNR with both B-and N-dopants, similar notation is used for the four vacancies-GNR. The normalization was done based on the case with non-dopants atoms in each group.

Before concluding, we compare the resonance frequencies and *Q*-factor among all testing samples. As seen in Figure 12a, the resonance frequency usually decreases with the increase of the dopant percentages, which is evidently seen in the perfect GNR with B-dopants. The largest reduction of the resonance frequency is observed in a perfect GNR with 2.65% B-dopant, about 14% decrease. It is worth to mention that, the B- and N-doped defective GNR with four vacancies (dopant densities of 0.26% and 2.40%) even exhibit a larger resonance frequency than their pristine counterpart. Further, the reduction of the resonance frequency for the two groups of doped defective GNR with four vacancies is much smaller than that of other groups. These observations suggest that the vacancies will also exert significant influence to the resonance properties of GNR. It is observed from Figure 12 that, the doping with only boron induces a larger reduction of the resonance frequency of either perfect or defective GNR than doping with both boron and nitrogen. Figure 12b compares the *Q*-factor obtained from different samples. Note that, some cases exhibit a nonlinear profile for the external energy (e.g., Figure 4a, Figure 9b), for which a valid *Q* cannot be estimated. Thus, these cases are not compared, as well as the two cases with significantly enhanced *Q* (i.e., GNR with 1.8% and 2.4% of B- and N-dopant). In general, the impacts from different percentages of dopants vary greatly. As is seen, most of the tested samples show a decreased *Q*, while some cases show an enhanced *Q*. It is assumed that the locations of dopant atoms also exert great influence on the *Q-*factor, and future works are expected to unveil such influence.

## Conclusion

Based on the MD simulations, we investigated the impacts of different dopants (only boron and boron with nitrogen) on the resonance properties of graphene nanoribbons (GNRs). Both perfect and defective (with either two or four randomly located vacancies) GNRs have been adopted as the samples, and the dopant densities were chosen below 3%. Major findings include: (a) Generally, the presence of dopants will lead to a degradation of the Q-factor and the resonance frequency of the GNR. (b) The impacts of doping on the *Q-*factor of perfect and defective GNR vary greatly, and there is no apparent correlation between the reduction of *Q* and the density of the dopant. (c) Compared with the influence on the *Q*-factor, the influences exerted on the resonance frequency are insignificant, i.e., the majority of the reduction is between 2–10%. (d) The reduction of resonance frequency of perfect and defective GNRs is larger when they are doped with boron only than when they are doped with both born and nitrogen. This study provides a comprehensive study of the impacts of different dopants on the resonance properties of graphene. The simulation techniques presented herein should also be applicable to graphene with other dopant elements (e.g., sulfur or silicon).
